# Effect of Substrate Composition on Bioconversion and Nutritional Quality of *Hermetia illucens* Larvae in Tropical Systems

**DOI:** 10.3390/insects17080799

**Published:** 2026-08-01

**Authors:** Ana M. E. Camargo García, Stephanie Samaniego, Gustavo Quirós, Pablo Montero-Prado

**Affiliations:** 1 Facultad de Ciencias y Tecnología, Universidad Tecnológica de Panamá, Panama City 0819-07289, Panama; ana.camargo3@utp.ac.pa (A.M.E.C.G.); stephanie.samaniego@utp.ac.pa (S.S.); 2 Centro de Innovación y Transferencia Tecnológica (CITT), Universidad Tecnológica de Panamá, Aguadulce 02038, Panama; gustavo.quiros2@utp.ac.pa; 3 Sistema Nacional de Investigación (SNI), National Secretariat of Science, Technology and Innovation, Panama City 0816-02852, Panama

**Keywords:** black soldier fly larvae, *Hermetia illucens*, organic waste valorisation, insect biomass, feed ingredient, circular bioeconomy

## Abstract

Organic waste valorisation using black soldier fly larvae (BSFL), *Hermetia illucens* L., offers a biological strategy to transform biodegradable residues into biomass with potential feed applications. This study evaluated five fruit–vegetable residue mixtures under tropical conditions: 100% fruit (100F), 100% vegetables (100V), 50% fruit–50% vegetables (50F–50V), 70% fruit–30% vegetables (70F–30V) and 30% fruit–70% vegetables (30F–70V), using three independent rearing units per diet. The analysis included initial egg mass, estimated viable larvae, coffee husk input as structural bulking material, tray-level process conditions, larval biomass, dry feed offered and estimated bioconversion indicators. The 100F treatment produced the greatest dry biomass (0.1311 ± 0.0132 kg), while 100V and 50F–50V showed favourable input-normalised responses. Biomass was also expressed relative to estimated viable larvae and dry feed offered to support treatment interpretation under the operational conditions evaluated. Coffee husk improved substrate handling and was incorporated into the dry-matter balance; therefore, adjusted BCR was interpreted as a process balance indicator and complemented with biomass yield per dry feed offered. Estimated protein values ranged from 35.4% to 38.1% in selected composite samples. *Salmonella* spp. were absent in pooled microbiological samples, *Escherichia coli* was <10 CFU/g, and screened heavy metals were below the laboratory detection limit in selected composite samples. Overall, the results support the potential of BSFL systems for valorising local fruit and vegetable residues under tropical conditions.

## Simple Summary

Black soldier fly larvae can transform organic residues into useful biomass for potential feed applications. Their performance, however, depends on the substrate-rearing conditions. This study compared five fruit–vegetable residue mixtures under tropical conditions and reports the initial egg mass, estimated viable larvae, and coffee-husk inputs used in each treatment. The fruit-only treatment generated the greatest total biomass per rearing unit. To support a balanced interpretation, biomass was also evaluated in relation to estimated viable larvae and dry feed offered. When biomass was normalised to estimated viable larvae and dry feed offered, the treatment ranking became less absolute. Selected composite biomass samples showed differences in protein, fat, energy and minerals. The results support the potential of BSFL for valorising local fruit and vegetable residues and identify key variables for future optimisation, including substrate characterisation, standardised initial density, survival monitoring and treatment-specific safety assessment.

## 1. Introduction

The increasing generation of organic residues represents a major challenge for sustainable food systems because large quantities of biodegradable material are still discarded without adequate recovery of their nutrients or productive value [1]. Fruit and vegetable residues are particularly relevant to this problem because they are continuously generated during distribution, retail, and consumption and are characterised by high moisture content and rapid deterioration [2]. At the same time, livestock and aquaculture production require reliable protein and energy for feed, while conventional ingredients such as soybean meal and fishmeal are associated with economic volatility and environmental concerns [3]. Consequently, biological strategies capable of transforming organic residues into nutritionally valuable biomass are increasingly relevant within circular bioeconomy models [4].

Black soldier fly larvae (BSFL), *Hermetia illucens* L. (Diptera: Stratiomyidae), have emerged as promising organisms for the bioconversion of organic residues [5]. During their larval stage, they can consume heterogeneous biodegradable substrates and transform part of the available nutrients into biomass containing protein, lipids and minerals [6,7]. The process also generates residual material with potential agronomic value, thereby linking waste management with alternative feed ingredients production [2,8].

BSFL performance depends strongly on the substrate supplied. Substrate moisture can influence residue separation, larval growth and survival [9], while substrate type and nutrient availability affect larval development and process efficiency [10,11]. In addition, different waste streams can modify larval performance, residual material generation and bioconversion outcomes [12]. Fruit and vegetable residues can differ substantially in botanical composition, moisture, fibre structure and soluble nutrient fractions; therefore, when real and heterogeneous waste streams are used, the responses observed should be interpreted in relation to the specific residue mixtures evaluated and the substrate characteristics reported.

Substrate formulation is also relevant because larval biomass is not a nutritionally invariant product. The protein, lipid and mineral composition of BSFL may be affected by the rearing substrate and larval development conditions [13]. Processing and rearing scale can also influence macronutrient composition and the final nutritional profile of the harvested biomass [14,15]. This variability is important when larval biomass is considered for animal feed applications, since its practical value depends not only on biomass production but also on nutritional characteristics and safety profile [2,5]. The use of organic residues also requires attention to microbiological quality and potential chemical contaminants [7,16].

Although BSFL bioconversion has been examined using food waste, agro-industrial by-products and plant-derived substrates [8,12], information remains limited on the response of BSFL to defined fruit-to-vegetable residue ratios under tropical conditions, particularly when real local waste streams are used. Previous studies have shown that fruit by-products, vegetable residues and plant-based food wastes can affect larval biomass yield, substrate reduction and biomass composition [10,17,18]. This knowledge gap is relevant for countries such as Panama, where warm environmental conditions and the availability of fruit and vegetable residues may favour the development of local BSFL-based valorisation strategies, while also influencing substrate stability, microbial dynamics and process management [19].

Accordingly, this study evaluated the effect of five diets formulated with different proportions of fruit and vegetable residues on the bioconversion performance of *H. illucens* larvae under tropical conditions. Larval biomass production, residual material generation, dry feed offered, estimated bioconversion indicators and process conditions were assessed. Selected nutritional composition indicators and microbiological and chemical safety parameters were evaluated descriptively. To support a balanced interpretation of treatment performance, the study also reports initial egg mass, estimated viable larvae, coffee-husk input as structural bulking material, and input-normalised biomass indicators.

## 2. Materials and Methods

### 2.1. Study Location and Biological Material

The experiment was conducted at the Centro de Innovación y Transferencia Tecnológica (CITT) of the Universidad Tecnológica de Panamá, Aguadulce, Coclé Province, Panama, at 29 m.a.s.l. (8°14′24″ N 80°32′24″ W). BSFL were obtained from eggs laid by captive adult *H. illucens* in the CITT pilot bioconversion facility; the colony originated from adults collected in the Aguadulce region.

### 2.2. Substrate Preparation and Experimental Diets

Fresh fruit and vegetable residues representative of locally generated organic discards from the Aguadulce region were selected and mechanically shredded to a maximum particle size of 1 cm using a blade shredder/biotriturator (Torotrac TR-200, TRAPP, Santa Catarina, Brasil).

Five diets were formulated on a fresh-weight proportion basis: 100F, 100V, 50F–50V, 70V–30V and 30F–70V (Table 1). The diets were designed with different fruit:vegetable proportions while maintaining the relative botanical composition within each fraction. The quantities reported correspond to a base formulation from which the experimental units were proportionally scaled. Percentages were calculated independently for the fruit and vegetable fractions.

The substrates consisted of real, non-standardised local waste streams. Moisture was determined for each substrate mixture during the experiment; complete proximate characterisation of the incoming diets was not included in the analytical design.

Egg mass was recorded for each experimental unit. The initial number of eggs was estimated from recorded egg mass using an egg mass-to-count conversion factor of approximately 39,100 eggs/g, and the number of viable larvae was estimated using a hatchability value of 82%. Treatment setup followed egg availability from the colony and the operational workflow of the pilot facility. Because recorded egg mass differed among treatments, with 100F receiving the highest egg input, biomass responses were reported both as total biomass per rearing unit and as biomass normalised to the estimated number of viable larvae. At harvest, total fresh larval biomass was recorded for each rearing unit.

Coffee husk was incorporated as a structural bulking material to improve substrate handling, porosity and consistency during rearing. The amount of coffee husk differed among treatments and is reported in Table 2. Its dry-matter contribution was calculated from its measured moisture content, which was approximately 10%.

**Table 2:** Experimental design and initial inputs used for each dietary treatment.

Parameter	100F	100V	50F–50V	70V–30V	30F–70V
Estimated egg mass (g/tray)	0.1560 ± 0.0003	0.1011 ± 0.0001	0.1012 ± 0.0002	0.1012 ± 0.0001	0.1012 ± 0.0001
Estimated viable larvae (tray^−1^)	5002 ± 8	3242 ± 3	3244 ± 5	3244 ± 2	3246 ± 2
Coffee husk (dry bulking material, kg/tray)	1.476 ± 0.000	1.116 ± 0.000	1.251 ± 0.000	0.981 ± 0.000	1.206 ± 0.000

Note. Values are mean ± standard deviation (n = 3). Estimated viable larvae were calculated from recorded egg mass using the egg mass-to-count conversion factor and hatchability value described in Section 2.2. Coffee husk was incorporated as structural bulking material, and values represent its dry-matter contribution calculated from the measured moisture content (approximately 10%). The experimental workflow is presented in Figure 1.

**Figure 1:**
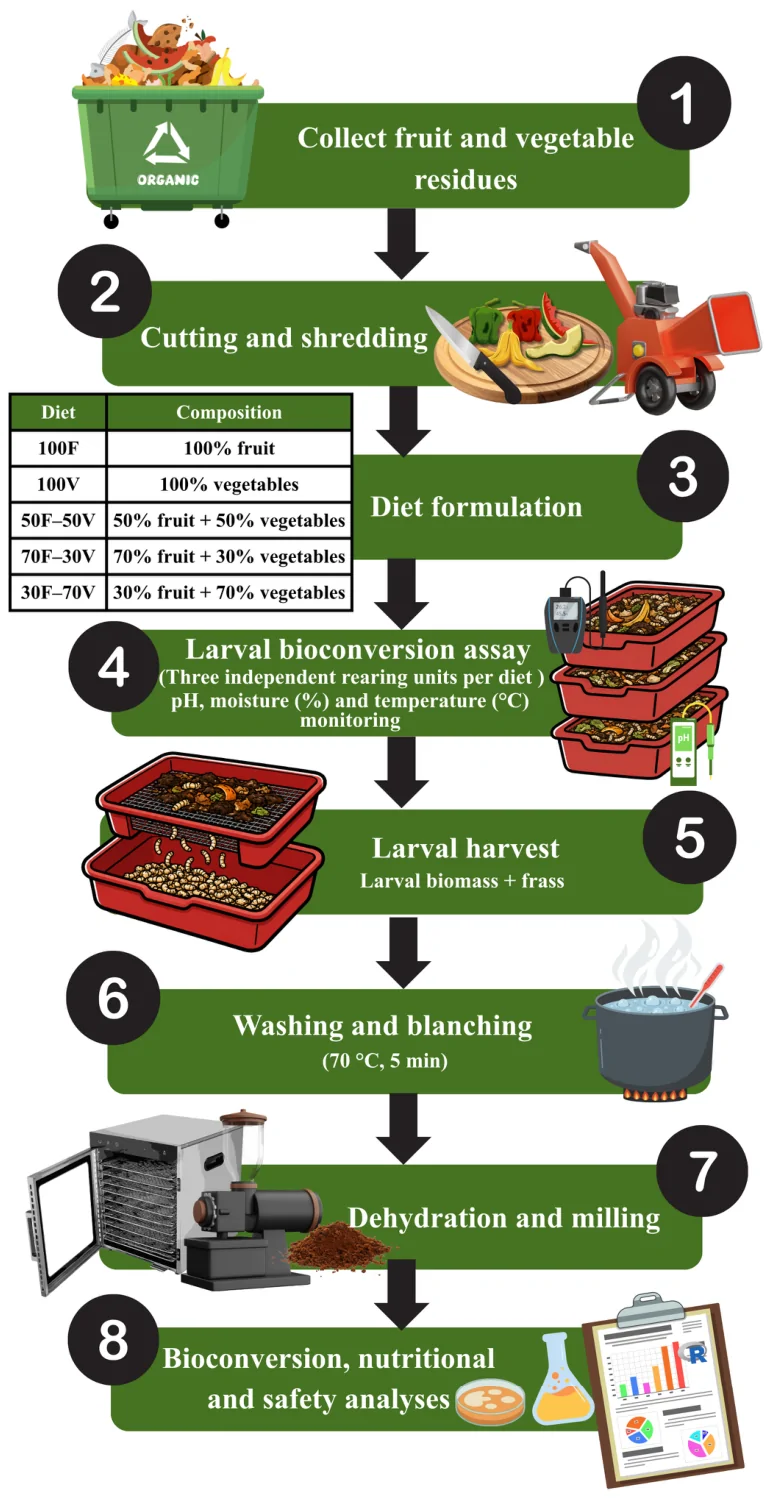
Experimental workflow for evaluating fruit–vegetable waste bioconversion by black soldier fly larvae (*Hermetia illucens*).

### 2.3. Experimental Design, Feeding and Monitoring

An exploratory completely randomised pilot design was used, with five diets and three independent rearing units per diet, giving a total of fifteen experimental units. Treatments were implemented sequentially according to egg availability from the colony and the operational workflow of the laboratory. The number of replicates was defined by colony size, available rearing space, processing capacity and the amount of biomass required for post-harvest analyses. Therefore, the study evaluated BSFL performance under the operational conditions of the pilot facility.

Feeding was standardised using a staged larval feeding rate on a fresh-matter basis: 45 mg/larva/day from days 0–5 after eclosion, 90 mg/larva/day from days 5–10 and 163 mg/larva/day from days 10–15. This feeding regime was applied to all treatments based on the estimated number of viable larvae in each rearing unit. Because the initial estimated larval number differed among treatments, the total amount of feed offered also varied accordingly. Temperature, substrate moisture and pH were measured repeatedly within each rearing unit during the larval period. For statistical comparisons among diets, the rearing tray was considered the independent experimental unit, and the temporal mean of each tray was used for variables measured repeatedly over time.

### 2.4. Harvest, Processing and Analytical Assessment

Larvae were harvested on days 15–16, before the prepupal stage, by manual separation using 5 mm mesh screens. They were washed with clean water to remove adhered residual material and then surface-drained using absorbent paper and an airflow-assisted draining step. The time between washing and the beginning of dehydration was approximately 30 min. After draining, larvae were weighed and transferred to the blanching and dehydration stages. The same harvest, washing, draining and pre-drying handling procedure was applied to all experimental units.

Larvae were then blanched at 70 °C for 5 min, dehydrated in a tray dehydrator (Avantco Equipment, Lititz, PA, USA) at 60 °C for 24 to 48 h, milled using a high-speed multi-function comminutor (model 1000C), vacuum-packed and stored at −18 °C until analysis. Final moisture was verified with an Ohaus MB90 (OHAUS Corporation, Shanghai, China) moisture analyser and was approximately 8–12%.

Physicochemical and microbiological analyses of the final larval biomass were performed by an authorised laboratory (Industrial Analysis Laboratory, S.A., Panama City, Panama, is accredited by government institutions, including the Ministry of Health (MINSA) under Certificate No. 341-C.T. 2009, and the National Environmental Authority (ANAM), as published in Official Gazette No. 25,059 of Thursday, 27 May 2004). Composite samples from the 100F, 50F–50V and 100V treatments were analysed for energy, protein, fat, total carbohydrates, phosphorus, calcium, iron, zinc and heavy metals. The laboratory reported crude protein using the conventional AOAC nitrogen-to-protein factor N × 6.25. For this manuscript, protein values were recalculated using an insect-specific nitrogen-to-protein conversion factor of N × 4.76, as proposed for insect biomass including *H. illucens* [20,21].

A global composite sample included biomass from all evaluated dietary treatments, was analysed for total aerobic bacteria, *Escherichia coli*, yeasts and moulds, and the presence or absence of *Salmonella* spp. Analytical methods were reported by the authorised laboratory as AOAC, 22nd edition (2023), for physicochemical and heavy metal determinations and BAM-AOAC, 17th edition, for microbiological analyses.

Based on the minimum sample quantity required by the authorised laboratory, biomass obtained from the three replicates within each selected treatment was pooled to generate one composite sample per treatment for physicochemical evaluation. Microbiological analyses were performed using one global composite sample integrating biomass from all evaluated dietary treatments. Therefore, nutritional composition and safety results were interpreted as descriptive indicators, and no inferential statistical comparisons were performed for these parameters.

### 2.5. Calculations of Bioconversion Indicators

Dry feed offered (DFO) was calculated from the cumulative amount of fresh substrate supplied to each rearing unit throughout the experimental period according to the staged feeding schedule described in Section 2.3. Fresh feed was converted to dry matter using the measured moisture content of each diet. Dry larval biomass and the remaining process-balance indicators were then calculated according to the following equations.

Dry larval biomass was calculated as:

$$DB=FB\times \left(1-\frac{LM}{100}\right)$$

where:

DB is dry larval biomass;

FB is fresh larval biomass;

LM is larval moisture (%).

Coffee husk dry matter was calculated as:

$$CH_{DM}=CH_{FW}\times \left(1-\frac{CH_{M}}{100}\right)$$

where:

CH_DM_ is coffee husk dry matter;

CH_FW_ is fresh coffee husk weight;

CH_M_ is coffee husk moisture (%).

Raw corrected residual material was calculated as:

$$RCR=DF-CH_{DM}$$

where:

RCR is raw corrected residue;

DF is measured dry frass.

Adjusted substrate consumption was calculated as:

AC=DFO−max(RCR,0)

where:

AC is adjusted consumption;

DFO is dry feed offered.

*Negative RCR values were set to zero.

Adjusted bioconversion rate was calculated as:

$$BCR_{adj}=\frac{DB}{AC}$$

Adjusted bioconversion efficiency was calculated as:

$$BCE_{adj}=BCR_{adj}\times 100$$

As a sensitivity metric, no-truncation BCR was calculated as:

$$BCR_{NT}=\frac{DB}{DFO-RCR}$$

Dry biomass yield per dry feed offered was calculated as:

$$Y_{DFO}=\frac{DB}{DFO}$$

### 2.6. Statistical Analysis

Experimental data were organised and analysed in R, version 4.6.0. The independent experimental unit was the rearing tray. For temperature, moisture and pH, repeated measurements were first averaged within each tray before treatment comparisons. Results are reported as mean ± standard deviation for the three independent experimental units per treatment. Normality and homogeneity of variance were assessed using Shapiro–Wilk and Levene tests, respectively. Variables meeting these assumptions were evaluated using one-way ANOVA followed by Tukey’s post hoc comparisons. When assumptions were not met, Kruskal–Wallis tests followed by Dunn comparisons were implemented when appropriate. Statistical significance was established at *p* < 0.05.

## 3. Results

### 3.1. Process Conditions and Initial Experimental Inputs

Experimental inputs and tray-level process conditions are shown in Table 3. The 100F treatment had the highest recorded egg mass and, consequently, the highest estimated number of viable larvae, consistent with the sequential establishment of treatments according to egg availability from the colony. Temperature remained within a narrow range across diets and did not differ significantly when tray-level means were analysed by ANOVA (*p* = 0.0556). Substrate moisture and pH differed significantly among diets, with 100F showing the most acidic mean pH and 100V the highest mean pH. Although the lowest pH values were recorded in the 100F treatment, no abnormal larval behaviour was visually observed during routine colony management. Final larval biomass was recorded at harvest; however, final larval counts and developmental rate were not included as experimental endpoints, so the possible effects of substrate acidification on survival or individual growth could not be assessed directly.

### 3.2. Larval Biomass, Residual Material and Bioconversion

Larval biomass, dry feed input and estimated bioconversion indicators are summarised in Table 4. The 100F treatment produced the greatest fresh and dry biomass per rearing unit. Because this treatment also had the highest estimated initial number of viable larvae, total biomass per tray was evaluated together with biomass normalised to estimated viable larvae and dry feed offered. When dry biomass was expressed per estimated viable larva, differences among treatments became less pronounced, indicating that treatment performance was influenced by both total biomass production and input-normalised responses.

Dry feed offered differed among treatments because feed allocation was calculated according to the estimated number of viable larvae in each rearing unit. Adjusted BCR was calculated using the corrected-residue procedure described in Section 2.5. Since this indicator depends on the dry-matter balance involving coffee husk as structural bulking material, the no-truncation BCR and dry biomass yield per dry feed offered were included as complementary indicators to support treatment comparison.

### 3.3. Composition and Preliminary Safety Assessment of Final Biomass

The descriptive physicochemical results for the three selected composite samples are presented in Table 5. Among the analysed samples, biomass from the 100V treatment showed the highest estimated protein value (38.1%) and the highest energy value (444 kcal/100 g), whereas biomass from the 50F–50V treatment showed the highest fat content (25.4%). Arsenic, lead, cadmium and mercury were below the laboratory detection limit in all three analysed treatment composites.

The microbiological assessment of one pooled sample combining biomass from all dietary treatment is shown in Table 6. *Salmonella* spp. were not detected in 25 g, and *Escherichia coli* was <10 CFU/g. The aerobic plate count and yeast and mould count provide process-hygiene information for the processed larval biomass. Because the analysis was performed on one pooled sample, these microbiological results are presented as descriptive safety indicators rather than treatment-level comparisons.

## 4. Discussion

This study showed that the five fruit–vegetable residue mixtures produced distinct BSFL performance patterns under the tropical experimental conditions evaluated. The 100F treatment generated the greatest total fresh and dry biomass per rearing unit. Because initial larval density differed among treatments, total biomass was interpreted together with biomass normalised to estimated viable larvae and dry feed offered. This combined interpretation provides a more complete view of treatment performance by considering both biomass production per unit and input-normalised responses.

Final larval biomass was determined by weighing the harvested larvae from each rearing unit. This endpoint allowed comparison of total fresh and dry biomass among treatments. Final larval counts and individual larval weights were not included as experimental endpoints; therefore, survival rate and individual growth could not be calculated. Future studies including final larval counts would allow a more detailed separation of survival, density and individual growth effects.

The use of coffee husk as a structural bulking material improved substrate handling and porosity and was incorporated into the dry-matter balance. Corrected residual material was calculated by subtracting the dry-matter contribution of coffee husk from the measured dry frass. For adjusted-BCR calculation, negative corrected-residue values were set to zero to avoid biologically inconsistent residual values. Since this correction affects adjusted consumption and adjusted BCR, these indicators were interpreted as process-balance estimates rather than direct measurements of substrate intake. Therefore, biomass yield per dry feed offered was also included as an input-based indicator to support comparison among treatments.

The responses observed in this study reflect the performance of BSFL reared on specific fruit–vegetable waste mixtures collected under local tropical conditions. Although the use of heterogeneous waste streams increases the practical relevance of the study by representing realistic organic waste generated in commercial and municipal settings, the absence of proximate characterisation of each substrate mixture limits the identification of the nutritional factors responsible for the observed responses. Consequently, differences among treatments cannot be attributed to specific nutritional characteristics such as protein, lipid, fibre or energy content, but rather to the overall residue mixtures evaluated. Fruit and vegetable residues can vary substantially according to botanical composition, maturity stage, storage conditions and seasonal availability, all of which may influence BSFL growth and bioconversion performance [9,10,11,12,17,18,22]. Therefore, the present findings should be interpreted as representative of the specific residue mixtures evaluated under the conditions of this study rather than as general responses to fruit- or vegetable-based waste streams. Future studies should include complete physicochemical characterisation of each substrate batch to improve reproducibility and facilitate comparisons among studies.

In this study, compositional analyses were performed on composite samples; therefore, the results are presented as descriptive indicators of product quality rather than as inferential comparisons among treatments.

The microbiological and chemical safety results provide an initial indication of the quality of the processed larval biomass. The absence of *Salmonella* spp. and the low *Escherichia coli* count in the pooled sample are favourable findings and align with the relevance of microbiological control in insect-based feed ingredients [7,23,24,25]. The non-detection of the screened heavy metals in selected composite samples indicates that these contaminants were not detected in the analysed batches, which is particularly relevant because chemical safety in BSFL systems depends strongly on substrate origin and quality [16,24]. At the same time, the aerobic plate count reinforces the need for controlled post-harvest handling, including hygienic processing, effective drying, appropriate storage, and routine verification when BSFL biomass is intended for feed applications [23,24,25]. Because safety outcomes in BSFL systems depend on both the substrate and the processing conditions, future assessments should include treatment-specific microbiological analyses and batch-level monitoring [7,23,24,25].

### Practical Implications

The findings of this study support the potential use of BSFL systems as an applied strategy for the valorisation of local fruit and vegetable residues under tropical conditions, consistent with previous studies on BSFL-based organic waste valorisation [1,2,7,8]. From a practical perspective, the results show that different fruit-vegetable residue mixtures can influence larval biomass production, residual material generation and the nutritional characteristics of the harvested biomass. This information can guide preliminary feeding strategies and help identify operational variables for small-scale and pilot BSFL systems.

Future implementation should incorporate substrate characterisation, standardised larval density, survival monitoring and validated dry-matter balance methods to improve process reproducibility and comparability. In addition, hygienic post-harvest processing, appropriate drying and storage conditions, and regulatory assessment should be considered before using BSFL biomass in feed-related applications [3,5,16,24,25]. Further studies including economic analysis, life-cycle assessment and feeding trials would support evaluation of industrial feasibility, cost-effectiveness, and environmental performance under tropical production scenarios.

## 5. Conclusions

Fruit–vegetable substrate formulation influenced process conditions, larval biomass production and estimated bioconversion indicators of *H. illucens* under tropical experimental conditions. The 100F treatment produced the greatest total fresh and dry biomass per rearing unit, while the 100V and 50F–50V treatments showed favourable input-normalised responses. These results indicate that both total biomass production and input-normalised indicators are useful for interpreting BSFL performance when initial larval density and substrate inputs differ among treatments. The 30F–70V treatment showed the lowest performance across several indicators.

Coffee husk functioned as a structural bulking material that improved substrate handling and was incorporated into the dry-matter balance. Adjusted consumption and adjusted BCR were interpreted as process-balance indicators and complemented by biomass yield per dry feed offered to support treatment comparison. Descriptive compositional results showed variation in the nutritional profile of the harvested biomass among the evaluated residue mixtures. The safety screening showed absence of *Salmonella* spp. in the pooled sample and no screened heavy metals above the laboratory detection limit in selected composite samples.

The findings support the potential of BSFL systems for the valorisation of local fruit and vegetable residues under tropical conditions. Future studies incorporating simultaneous treatment establishment, standardised larval density, final survival counts, complete substrate characterisation, validated residue-recovery methods and replicated treatment-specific nutritional, and safety analyses would strengthen process reproducibility and support future scale-up or feed-related applications.

## Figures and Tables

**Table 1:** Botanical composition of the experimental diets (% within each fruit or vegetable fraction).

Ingredient	100F	100V	50F–50V	70V–30V	30F–70V
Overall fruit proportion (%, *w*/*w*)	100	0	50	70	30
Overall vegetable proportion (%, *w*/*w*)	0	100	50	30	70
Fruit fraction					
Watermelon	25.0	—	10.3	10.3	9.8
Melon	21.8	—	26.8	26.8	—
Papaya	29.9	—	27.1	27.1	6.0
Pineapple	23.3	—	—	—	17.8
Green apple	—	—	5.7	5.6	—
Red apple	—	—	4.3	4.3	—
Orange	—	—	12.4	12.4	—
Tomato	—	—	13.4	13.4	19.9
Lemon	—	—	—	—	27.2
Chilli pepper	—	—	—	—	3.4
Cucumber	—	—	—	—	7.5
Zucchini	—	—	—	—	4.6
Chayote	—	—	—	—	3.9
Vegetable fraction					
Lettuce	—	52.3	20.0	20.0	52.3
Cabbage	—	38.3	26.0	26.0	38.3
Celery	—	4.7	8.8	8.8	4.7
Broccoli	—	4.7	24.6	24.6	4.7
Culantro	—	—	12.3	12.3	—
Onion	—	—	8.4	8.4	—

Note. Values represent the percentage contribution of each ingredient within the corresponding fruit or vegetable fraction used to formulate the experimental diets. Overall fruit and vegetable proportions are shown at the top of the table.

**Table 3:** Tray-level process conditions recorded during the larval rearing period.

Parameter	100F	100V	50F–50V	70V–30V	30F–70V
Temperature (°C)	28.78 ± 0.18	28.80 ± 0.07	28.98 ± 0.06	28.73 ± 0.11	28.96 ± 0.05
Moisture (%)	87.73 ± 0.49 ^a^	86.67 ± 0.16 ^a^	88.43 ± 0.31 ^a^	87.07 ± 0.31 ^ab^	85.05 ± 0.30 ^b^
pH	4.17 ± 0.07 ^c^	4.92 ± 0.04 ^a^	4.38 ± 0.07 ^b^	4.37 ± 0.06 ^b^	4.13 ± 0.06 ^b^

Note. Values are mean ± standard deviation (n = 3). Different superscript letters within the same row indicate significant differences among diets according to Tukey’s HSD test (*p* < 0.05).

**Table 4:** Larval biomass, dry feed input and estimated bioconversion indicators.

Diet	Fresh Biomass (kg)	Dry Biomass (kg)	Dry Biomass (mg/Viable Larva)	Dry Feed Offered (kg)	Raw Corrected Residue (kg)	Adjusted BCR (Truncated)	No-Truncation BCR	Dry Biomass/Dry Feed Offered
100F	0.448 ± 0.053	0.1311 ± 0.0132 ^a^	26.21 ± 2.68 ^ab^	1.270 ± 0.055 ^a^	−0.367 ± 0.053	0.1033 ± 0.0110 ^ab^	0.0800 ± 0.0073	0.1033 ± 0.0110 ^ab^
100V	0.309 ± 0.025	0.0917 ± 0.0037 ^b^	28.28 ± 1.14 ^a^	0.818 ± 0.067 ^d^	−0.015 ± 0.149	0.1199 ± 0.0039 ^a^	0.1106 ± 0.0080	0.1128 ± 0.0130 ^a^
50F–50V	0.307 ± 0.012	0.0905 ± 0.0051 ^b^	27.88 ± 1.58 ^ab^	0.822 ± 0.014 ^d^	−0.532 ± 0.020	0.1101 ± 0.0060 ^a^	0.0668 ± 0.0035	0.1101 ± 0.0060 ^ab^
70V–30V	0.236 ± 0.012	0.0697 ± 0.0047 ^b^	21.50 ± 1.43 ^ab^	0.956 ± 0.042 ^c^	−0.133 ± 0.049	0.0732 ± 0.0073 ^bc^	0.0640 ± 0.0034	0.0732 ± 0.0073 ^bc^
30F–70V	0.187 ± 0.082	0.0560 ± 0.0262 ^b^	17.26 ± 8.09 ^b^	1.087 ± 0.026 ^b^	−0.038 ± 0.061	0.0523 ± 0.0246 ^c^	0.0499 ± 0.0225	0.0518 ± 0.0246 ^c^

Note: Values are mean ± standard deviation (n = 3). Different superscript letters within the same column indicate significant differences at *p* < 0.05 according to Tukey’s HSD. Corrected residue was calculated as measured dry frass minus the dry-matter contribution of coffee husk, which was calculated from its measured moisture content. For adjusted-BCR calculation, negative corrected-residue values were set to zero to avoid biologically inconsistent residual values. Adjusted BCR is presented as a process-balance indicator and is complemented by biomass yield per dry feed offered and no-truncation BCR as input-based comparison metrics.

**Table 5:** Physicochemical composition and heavy-metal screening of selected composite BSFL samples.

Parameter	Unit	100F	50F–50V	100V
Energy	kcal/100 g	436	428	444
Estimated protein	%	35.9	35.4	38.1
Fat	%	18.2	25.4	20.6
Total carbohydrates	%	21.0	13.6	14.6
Phosphorus	mg/100 g	972.5	884.6	992.4
Calcium	mg/100 g	1528.9	1923.0	1926.8
Iron	mg/kg	223.5	271.3	99.1
Zinc	mg/kg	74.1	75.0	77.3
Arsenic	-	<LOD	<LOD	<LOD
Lead	-	<LOD	<LOD	<LOD
Cadmium	-	<LOD	<LOD	<LOD
Mercury	-	<LOD	<LOD	<LOD

Note: Each value represents one composite sample obtained by pooling the three experimental replicates of the selected diet. LOD = limit of detection reported by the analytical laboratory. Protein values were calculated using the insect-specific nitrogen-to-protein conversion factor N × 4.76 [20,21].

**Table 6:** Preliminary microbiological assessment of pooled final BSFL biomass.

Parameter	Unit	Result
Aerobic plate count	CFU/g	5.5 × 10^6^
*Escherichia coli*	CFU/g	<10
Yeasts and moulds	CFU/g	1.9 × 10^2^
*Salmonella* spp.	in 25 g	Absent

Note: The microbiological results correspond to one global composite sample prepared by pooling larval biomass from all evaluated dietary treatments. Therefore, the values are presented as descriptive safety and process-hygiene indicators and are not intended for treatment-level comparison. CFUs = colony-forming units.

## Data Availability

The data supporting the findings of this study are available from the corresponding author upon reasonable request.

## Abbreviations

The following abbreviations are used in this manuscript:

AC	Adjusted substrate consumption
ANOVA	Analysis of variance
AOAC	Association of Official Analytical Chemists
BAM	Bacteriological Analytical Manual
BCE	Bioconversion efficiency
BCE_adj	Adjusted bioconversion efficiency
BCR	Bioconversion rate
BCR_adj	Adjusted bioconversion rate
BCR_NT	No-truncation bioconversion rate
BSFL	Black soldier fly larvae
CFU	Colony-forming unit
CH_DM	Coffee husk dry matter
CH_FW	Fresh coffee husk weight
CH_M	Coffee husk moisture
DB	Dry larval biomass
DF	Measured dry frass
DFO	Dry feed offered
DM	Dry matter
FB	Fresh larval biomass
HSD	Honestly significant difference
LM	Larval moisture
LOD	Limit of detection
N	Nitrogen
pH	Potential of hydrogen
RCR	Raw corrected residue
Y_DFO	Dry biomass yield per dry feed offered
